# Aquaporin 3 promotes human extravillous trophoblast migration and invasion

**DOI:** 10.1186/s12958-021-00726-z

**Published:** 2021-03-29

**Authors:** Yingqi Nong, Shifen Li, Wenjuan Liu, Xiqian Zhang, Lin Fan, Ye Chen, Qianwen Huang, Qianyu Zhang, Fenghua Liu

**Affiliations:** 1 The First Affiliated Hospital of Jinan University, Guangzhou, China; 2 Department of Reproductive Health and Infertility, Guangdong Women and Children Hospital, Guangzhou, Guangdong China; 3 Reproductive Medicine Center, Affiliated Shenzhen City Maternity and Child Healthcare Hospital of Southern Medical University, Shenzhen, China

**Keywords:** Aquaporin 3 (AQP3), Embryo implantation, Human extravillous trophoblast, Migration, Invasion

## Abstract

**Problem:**

Does aquaporin 3 (AQP3) affect the migration and invasion of human extravillous trophoblast (HTR8/Svneo) cells?

**Method of study:**

A lentivirus infection system was used to construct stable cell lines with either AQP3 knockdown or overexpression. RT-PCR and western blotting were used to verify the efficiencies of AQP3 knockdown or overexpression in HTR8/Svneo cells at mRNA and protein levels, respectively. Cell Counting Kit-8 and flow cytometry assays were used to detect the influence of AQP3 knockdown or overexpression on proliferation and apoptosis of HTR8/Svneo cells. In addition, wound healing and Transwell invasion assays were used to detect the effects of AQP3 knockdown or overexpression on migration and invasion capabilities of HTR8/Svneo cells. An Agilent gene chip was used to screen for significant differentially expressed genes after AQP3 knockdown. Finally, mechanisms by which AQP3 influences the migration and invasion of HTR8/Svneo cells were explored using bioinformatic analysis.

**Results:**

Compared with controls, migration and invasion capabilities of HTR8/Svneo cells were significantly reduced after AQP3 knockdown, and significantly increased after AQP3 overexpression. Subsequent bioinformatic analysis of gene chip expression profiles indicated downregulation of genes related to adhesion such as PDGF-B, as well as signaling pathways (such as PIK3/AKT, NF-κB, and TNF) after AQP3 knockdown.

**Conclusions:**

AQP3 could significantly promote migration and invasion capabilities of human extravillous trophoblasts, it may mediate embryo invasion and adhesion to endometrium by regulating PDGF-B, PIK3/AKT signaling pathways, although this requires further verification.

**Supplementary Information:**

The online version contains supplementary material available at 10.1186/s12958-021-00726-z.

## Introduction

Recurrent implantation failure (RIF) is one of the bottlenecks of in vitro fertilization-embryo transfer and its derivative techniques. Currently, there is no uniform definition on RIF. However, it is widely accepted as a diagnosis criteria which include an age less than 40 years and failure to achieve a clinical pregnancy after transfer of at least four good-quality embryos in a minimum of three fresh or frozen cycles [1]. Incidence of RIF is up to 10–15% [2], but its pathogenesis is still unclear. Many studies have reported that two-thirds of RIF cases can be attributed to endometrial receptivity, while the other third is caused by inherent factors within embryos [3].

Embryo implantation is an important step of mammalian reproduction, and is critical for determining pregnancy. Two factors that determine embryo implantation are the embryo’s implantation capability and the receiving status of the endometrium. The process of embryo implantation includes localization, adhesion, and invasion of the maternal endometrium until embedment into the matrix. This behavior of the embryo invading the endometrium at a specific time and space is the primitive motive for embryo implantation. The invasion process is completed when EVT differentiate from cytotrophoblasts [4]. Decidualization happens in the endometrium during the implantation window after blastocyst adhesion. Endometrial interstitial cells gradually transform into decidual stromal cells, which receive the invasion of EVT [5]. It has been shown that invasion of trophoblasts into the endometrium is similar to the metastasis of malignant tumors [6], as both involve cell invasion. Interestingly, an in vitro co-culture study reported that trophoblasts in mouse embryos exhibited stronger invasion than malignant tumor cells [7, 8]. Directional migration of trophoblasts, which involves a series of cell signaling events, is a central step of invasion behavior. At present, widely accepted cell migration mechanisms [9] include: (1) actin depolymerization, transport of ions into cells, and increased osmotic pressure in the front of cells; (2) penetration of water through the cell membrane to increase local hydrostatic pressure, whereby the cell membrane forms local crowning including ruga, pseudopod, and vesicles; and (3) actin re-polymerization. Thus, migration speed can be controlled by osmotic pressure in extracellular medium. This means that high osmotic pressure accelerates migration, while low pressure slows down migration. During this process, AQPs play a critical role for compliance with the acceleration of intracellular and extracellular osmotic pressure changes, as well as rapid changes of cellular morphology [10, 11].

AQPs, a type of channel protein, can regulate levels of water and small molecular substances (such as glycerin, urea, and nitrogen), which is very important for the maintenance of body fluid equilibrium [12]. Multiple AQPs have been detected in embryos before implantation [12–14]. Expression of AQP3 lasts from the zygote to the blastula stage [15], and is the most abundant aquaporin expressed in villi during early pregnancy [16]. This suggests that AQP3 may play a role in early growth and implantation of embryos.

Our previous study found high expression of AQP3 in the cell membrane of trophoblasts in blastocysts of Kunming mice [17]. AQP3 could significantly promote both adhesion and expansion capabilities of blastocysts [18], suggesting that AQP3 participates in the process of trophoblasts invading the endometrium. However, it is unknown if AQP3 is expressed in human extravillous trophoblasts (EVT), or whether it participates in human embryo implantation. If so, the mechanism by which AQP3 regulates embryo implantation is unknown. All of these issues were addressed in the current study.

## Methods and materials

### Methods

#### Cell culture

The human trophoblast cell line HTR8/SVneo has been widely applied as an early invasion and migration model of extravillous cytotrophoblasts [19]. HTR8/SVneo, which was provided by American Type Culture Collection (USA), was cultured with Dulbecco’s Modified Eagle’s Medium (DMEM) containing 10% fetal bovine serum (FBS; Gibco, USA) at 37 °C and 5% CO_2_.

#### Construction of stable cell lines

Plasmids containing AQP3 knockdown, AQP3 overexpression, or their respective negative control plasmids were purchased from Weijiang Biotechnology (China). Lentiviral packaging was performed in 293 T cells according to the manufacturer’s instructions (Suzhou GenePharma, China). Lentivirus in the supernatant was collected to transfect cells. Controls were transfected with empty vector. Cells were divided into four groups: interfering AQP3 (AQP3-shRNA) group, interfering empty vector (CON-shRNA) group, overexpressing AQP3 (AQP3-OE) group and overexpressing empty vector (CON-OE) group. Cells were infected with viruses at the following multiplicities of infection (MOI): AQP3-shRNA (MOI =100); CON-shRNA (MOI =100); AQP3-OE (MOI =150); CON-OE (MOI =100). Strict phenotype selection was performed on stably infected HTR8/SVneo cells with 0–10 μg/mL puromycin (MPbio, USA) to use resistance as a screening index. Furthermore, cells were stably cloned in 0.5 μg/mL puromycin.

#### RNA isolation, cDNA synthesis and qRT-PCR analysis

Total RNA was extracted from HTR8/Svneo cells according to the instructions of a TRIzol kit (Taraka Biotechnology, China), and measured with a spectrophotometer (Nanodrop 2000; Thermo Scientific, USA).

##### cDNA synthesis

The annealing mixture contained 1 μg of RNA, 1 μl of 0.5 μg/ul Oligo (dT) 18, 1 μl of dNTPs Mix (2.5 mM), and RNase-free water to a total volume of 10 μl. Then, it was incubated in a thermal cycler at 65 °C for 5 min and placed on ice for 1 min at least. The contents of the tube were collected by brief centrifugation before the following were added to the tube: Random primer, 1 μl, 5 × Buffer, 4 μl, RNase Inhibitor, 0.5 μl, and RNase Free H2O 4.5 μl. The pipette was gently sucked and tapped several times to obtain a better mixture. Incubation was done as follows: 10 min at 30 °C, 60 min at 42 °C and then 15 min at 70 °C. The cDNA synthesis reaction was stored at − 20 °C, or PCR was performed immediately.

RT-qPCR reactions were conducted with a QuantStudio 5 real-time PCR system (Thermo Fisher Scientific, Waltham, MA USA). The primers were designed by Primer-BLAST (NCBI). The primer sequences and PCR product sizes were detailed in Table 1. The real time PCR mixture contained SYBR Green Premix, 10 μl, Forward Primer (10 μM), 0.4 μl, Reverse Primer (10 μM), 0.4 μl, Template,1.2 μl, ROX Reference Dye II (50×), 0.4 μl, DNA template 2.0 μl, and RNase Free H2O to a total volume of 20 μl. After the solution was mixed and centrifuged at 5000 RPM for a short time, the reaction mixtures (8 μl) were added into a 384-well PCR plate, and the cDNA samples (2 μl) were added. The plate was sealed and placed on ice. The PCR was initiated by heating the mixture to 95 °C for 5 min, followed by 40 cycles of 15 s at 95 °C and 60s at 60 °C and then 15 s at 95 °C. To establish the melting curve, the mixture was heated to 95 °C for 10 s, 60 °C for 60 s, and 95 °C for 15 s sequentially after the amplification reaction was over. The 2^-ΔΔCT^ method was used to quantify relative expression of AQP3 mRNA. Each real-time PCR included a no-template control. The experiments were repeated 3 times with triplicates of each sample.

**Table 1:** The Primer sequences and the product size of target and control genes

Primer	Primer sequence (5′-3′)	size (bp)
**AQP3**	Forward primer: ACCATCAACCTGGCCTTTGG Reverse primer: GGGGACGGGGTTGTTGTAG	390
**PDGFB**	Forward primer: ACTGATGGGGTCGCTCTTTG Reverse primer: CAGGGATCAGGCAGGCTATG	126
**FOS**	Forward primer: GTGCCAACTTCATTCCCACG Reverse primer: GGCCTCCTGTCATGGTCTTC	186
**SNAIL1**	Forward primer: CCTGTCTGCGTGGGTTTTTG Reverse primer: ACCTGGGGGTGGATTATTGC	198
**GAPDH**	Forward primer: GAAGCTCATTTCCTGGTATGACA Reverse primer: GGGAGATTCAGTGTGGTGGG	189

#### Western blotting

Cells were collected and lysed with RIPA lysis buffer and phenylmethylsulfonyl fluoride (Beyotime Biotechnology, China) on ice for 30 min, quantified by bicinchoninic acid assay. Equal amounts of protein (~ 10 μg) were separated by 10% sodium dodecyl sulfate polyacrylamide gel electrophoresis (SDS-PAGE) and loaded for SDS-PAGE electrophoresis. After transfer to a polyvinylidene fluoride membrane, the membrane was blocked in 5% skim milk, sealed for 1 h at room temperature with shaking, and incubated with a primary antibody over-night at 4 °C. The primary antibodies used were rabbit polyclonal anti-AQP3 antibody (1:1000, Abcam, UK, ab125219), rabbit polyclonal anti-PDGFB antibody (1:1000, Cohesion, UK, CPA1865). IgG from rabbit serum (1:1000, Sigma, USA, I5006) was used for negative control. Finally, the membrane was incubated with horseradish peroxidase-labeled secondary antibody (120,000, Boster Biological Technology, China, BA1054) at room temperature for 1 h, and developed by enhanced chemiluminescence. The experiments were repeated 3 times. Image Pro-Plus 6.0 software (Media Cybernetics, USA) was used to analyze gray values.

#### Cell proliferation/CCK-8 assay

HTR8/Svneo cells (100 μL; 1 × 10^5^ cells/mL) in vector control, knockdown, and overexpression groups were added into 96-well plates in triplicate, and cultured at 37 °C overnight. CCK-8 kit reagent (10 μL; Dojindo, Japan) was added into each well and incubated for 2 h, 3 h, and 4 h. Optical density at 450 nm of each well was detected each time point with a multifunctional microplate reader.

#### Flow cytometry assay

Annexin V-APC/7-AAD double staining was performed on HTR8/Svneo cells according to the instructions of an Annexin V-APC/7AAD Apoptosis Detection Kit [Multisciences (Lianke) Biotech, China]. Live, early apoptotic, and late apoptotic or necrotic cells were classified using flow cytometry (AccuriC6, Becton Dickinson, USA) and Flow Jo 7.6.1 software (FlowJo, USA).

#### Wound healing assay

HTR8/Svneo cells (1 × 10^6^ cells/mL) were seeded in a six-well plate and routinely cultured in an incubator. When cells grew into a monolayer, they were treated with mitomycin for 1 h to inhibit cell division. Next, a sterile 10 μl-pipette tip was used to scrape cell culture plates. Scraped cells were washed twice with phosphate-buffered saline, cultured in serum-free medium, incubated in an incubator, and photographed at 0, 6, 24 and 48 h after scratching. Image Pro-Plus 6.0 was used to measure scratch depth at any five sites at the same time point to calculate migration rates, thus reflecting cell mobility and migratory capabilities.

#### Transwell invasion assay

A Transwell invasion system (8-μm, 24-well; Corning, UK) coated with Matrigel (40 μL; Becton Dickinson) was used. Briefly, 1 × 10^5^ cells were suspended in DMEM without serum, and seeded in the upper chamber. DMEM containing 10% FBS was then added to the lower chamber and the plate was incubated at 37 °C and 5% CO_2_. After 24 h, cells were fixed with methanol and stained with 0.1% crystal violet. The quantity of colored cells in five random visual fields was counted using an inverted microscope (Nikon, Japan).

#### Whole genome expression profile

The Gene expression profiles assay was performed according to a previously described method [20]. Gene expression profiles of AQP3-shRNA and CON-shRNA were analyzed by two-color gene expression microarray (Agilent Technologies, USA) according to the instructions of a Low Input Quick Amp Labeling Kit Two-Color (Agilent). Total RNA obtained in the extraction phase was used as a template, and the first strand of cDNA was reverse transcribed using T7 RNA polymerase. The second strand of cDNA was used as the synthesis template to perform in vitro transcription and promote generation of cRNA. An Agilent cRNA labeling kit was used to incorporate cRNA with Cy-3, which allowed purification and qualification of cRNA (Nanodrop 2000). After hybridization, washing, and chip scanning, data were extracted to perform bioinformatic analysis using Agilent Feature Extraction Software. Doing q-PCR verfication for FDGF-B, FOS and Snail1, which showed significantly decrease in the results of the gene expression profile experiment.

#### Statistical analysis

Data were analyzed by SPSS 23.0 software (IBM, USA). Each experiment was performed in triplicate, and data were expressed as mean ± standard deviation (SD). Migration rate and invasion index were analyzed using a two independent-samples t-test. Proliferation and apoptosis rates were analyzed by analysis of variance. *P* <  0.05 was considered statistically significant.

## Results

### Verification of AQP3 knockdown and overexpression efficiency

**Fig. 1:**
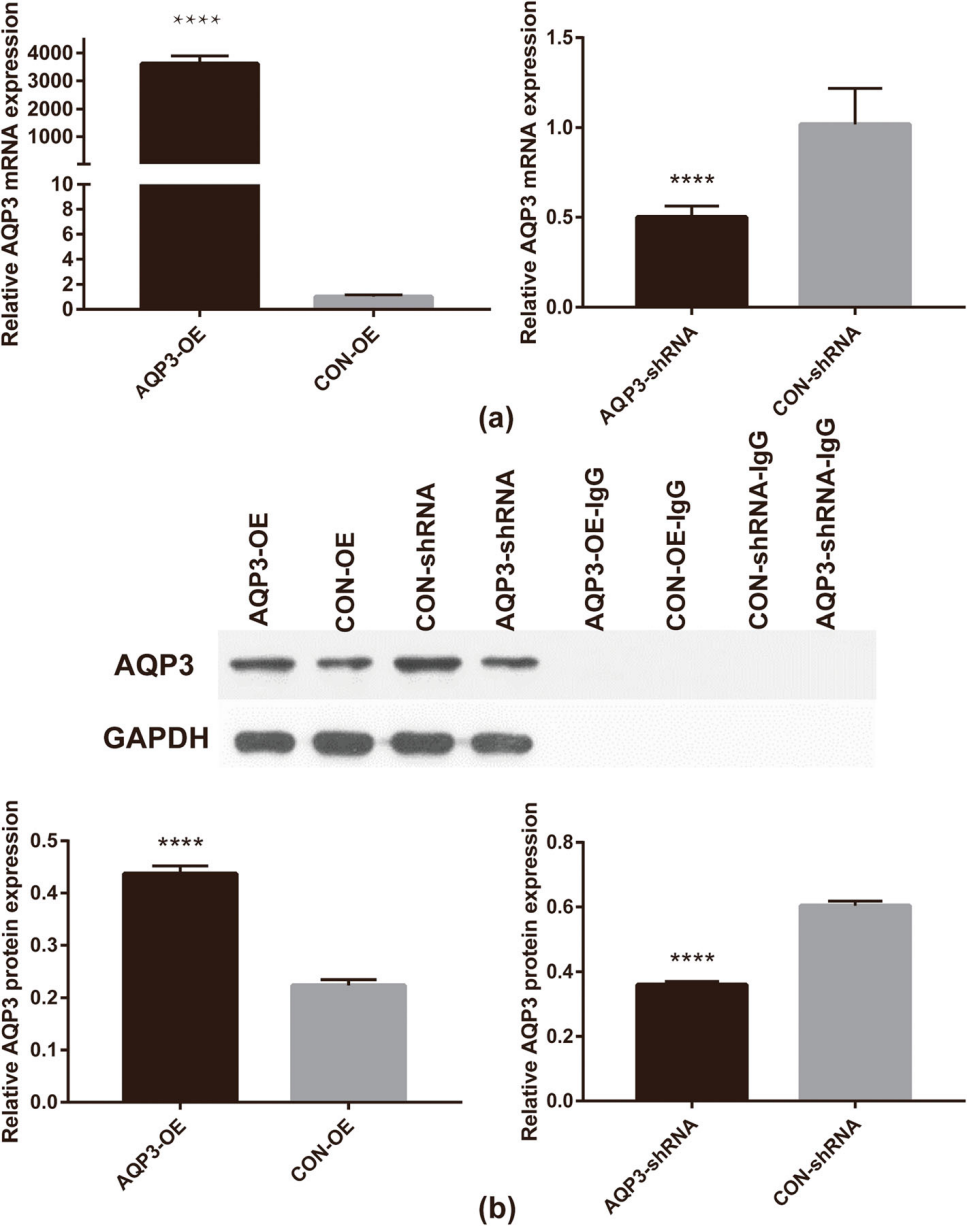
Verification of AQP3 knockdown and overexpression efficiency. (**a**) Relative expression levels of AQP3 mRNA in AQP3 knockdowned and overexpressed HTR8/SVneo. (**b**) Relative expression levels of AQP3 protein in AQP3 knockdowned and overexpressed HTR8/SVneo. Suggesting effective AQP3 knockdown or overexpression in HTR8/SVneo. **P < 0.01; ****P < 0.0001

After construction of stable cell lines, AQP3 knockdown and overexpression efficiencies at mRNA and protein levels in HTR8/Svneo cells were detected using RT-PCR and western blotting, respectively. The results indicated that compared with the CON-shRNA group, AQP3 knockdown resulted in downregulation of AQP3 mRNA expression by 50.8% (*P* < 0.0001), and downregulation of AQP3 protein levels by 40.3% (*P* < 0.0001). Compared with the CON-OE group, AQP3 overexpression resulted in upregulation of AQP3 mRNA expression by 3579-fold (*P* < 0.0001), and upregulation of AQP3 protein levels by 2-fold (*P* < 0.0001) (Fig. 1).

### AQP3 knockdown expression increased the apoptosis of HTR 8/SVneo cells but had no significant effect on their cell proliferation

**Fig. 2:**
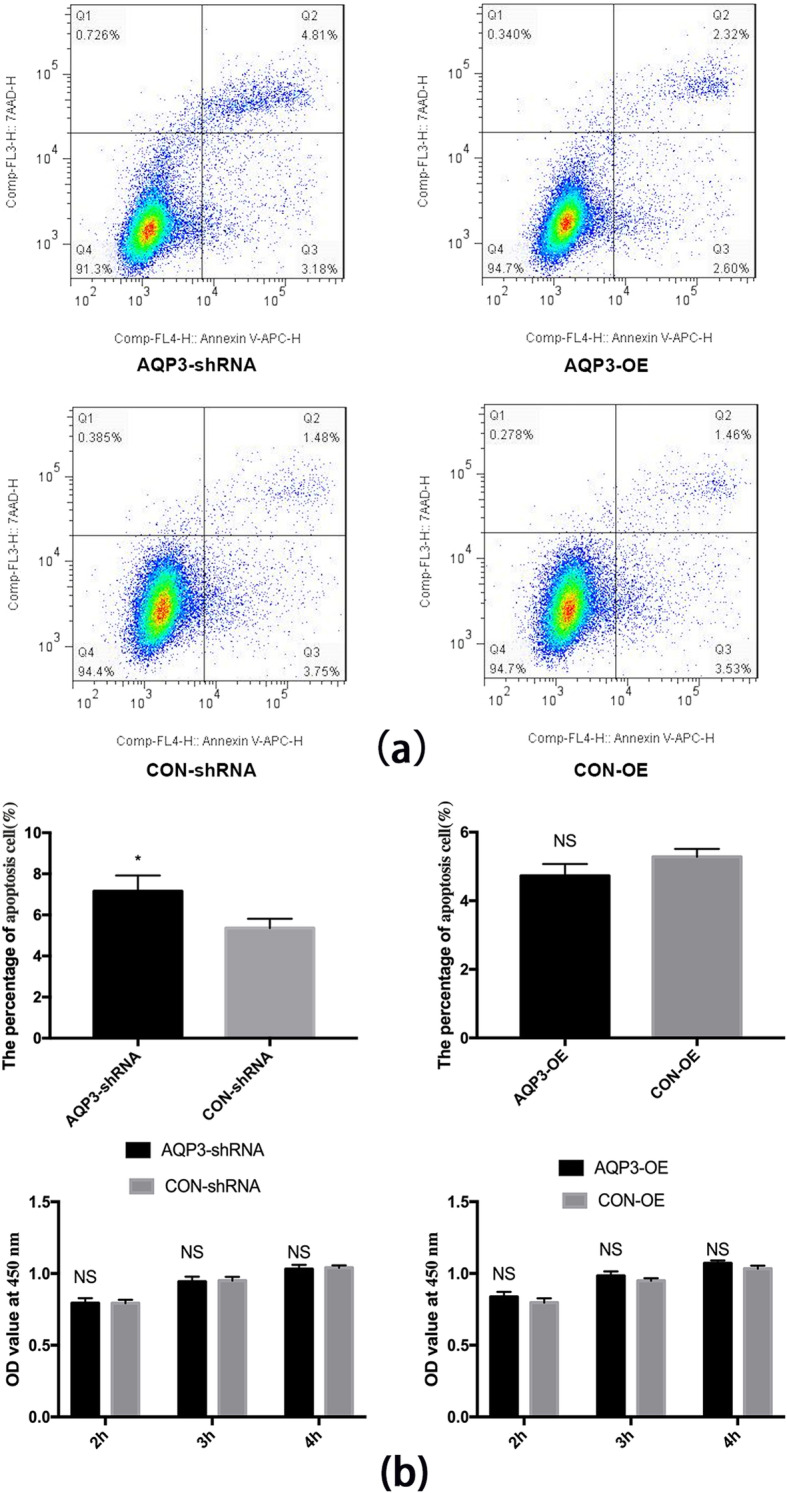
Flow cytometry and CCK-8 assays. (**a**) Apoptosis rates of HTR8/SVneo cells as analyzed by flow cytometry. (**b**) Proliferation rates as detected by CCK-8 assay. AQP3 knockdown expression increased the apoptosis of HTR 8/SVneo, but had not effect on cell proliferation. Overexpression of AQP3 had no effect either on the cell apoptosis or proliferation rates. * *P* < 0.05, NS:*P* >  0.05

Apoptosis rates of HTR8/SVneo cells were analyzed by flow cytometry while proliferation rates were detected by CCK-8 assay. Q2 (APC+/7AAD+) indicates late apoptotic/necrotic cells, Q3 (APC+/7AAD–) indicates early apoptotic cells, Q4 (APC−/7AAD–) indicates living cells. The apoptotic cells include late apoptotic and early apoptotic cells (Q2 + Q3). Compared with the control, apoptosis in the AQP3-shRNA group was significantly upregulated by 33.5% (*P* < 0.05). However, the rate of apoptosis in HTR8/Svneo cells in the AQP3-OE group was downregulated by 11.8% (*P* > 0.05) (one-way ANOVA) (Table 2, Fig. 2a). Proliferation rates in AQP3-shRNA, CON-shRNA, AQP3-OE, and CON-OE groups at 2 h,3 h,4 h after CCK-8 addition were not significantly different (*P* > 0.05). (two-way ANOVA). (Table 3, Fig. 2b).

**Table 2:** Apoptosis rates of HTR8/SVneo cells. The apoptotic cells include late apoptotic (Q2:APC+/7AAD) and early apoptotic cells (Q3:APC+/7AAD–)

Group	Apoptosis rates (Mean ± SEM)	***p***-value
AQP3-shRNA	7.157% ± 4.391%	< 0.05
CON-shRNA	5.36% ± 2.594%	
AQP3-OE	4.727% ± 1.984%	> 0.05
CON-OE	5.28% ± 0.1353%

**Table 3:** Proliferation rates of HTR8/SVneo cells

Time (after CCK-8 addition)	Proliferation rates (Mean ± SEM)		p-value
	AQP3-shRNA	CON-shRNA	
2 h	79.3% ± 3.5%,	79.3% ± 2.3%	> 0.05
3 h	94.3% ± 3.5%	95.0% ± 2.6%	> 0.05
4 h	100.3 ± 3.0%	100.4% ± 1.7%	> 0.05
**Time (after CCK-8 addition)**	**Proliferation rates (Mean ± SEM)**		**p-value**
	AQP3-OE	CON-OE	
2 h	83.7% ± 3.5%	79.7% ± 3.1%	> 0.05
3 h	98.3% ± 3.1%	95.0% ± 1.7%	> 0.05
4 h	100.70% ± 2.0%	100.3 ± 2.1%	> 0.05

### Migratory rate and invasive ability of HTR8/Svneo cells were increased after overexpressing AQP3 while be reduced after knockdown of AQP3

**Fig. 3:**
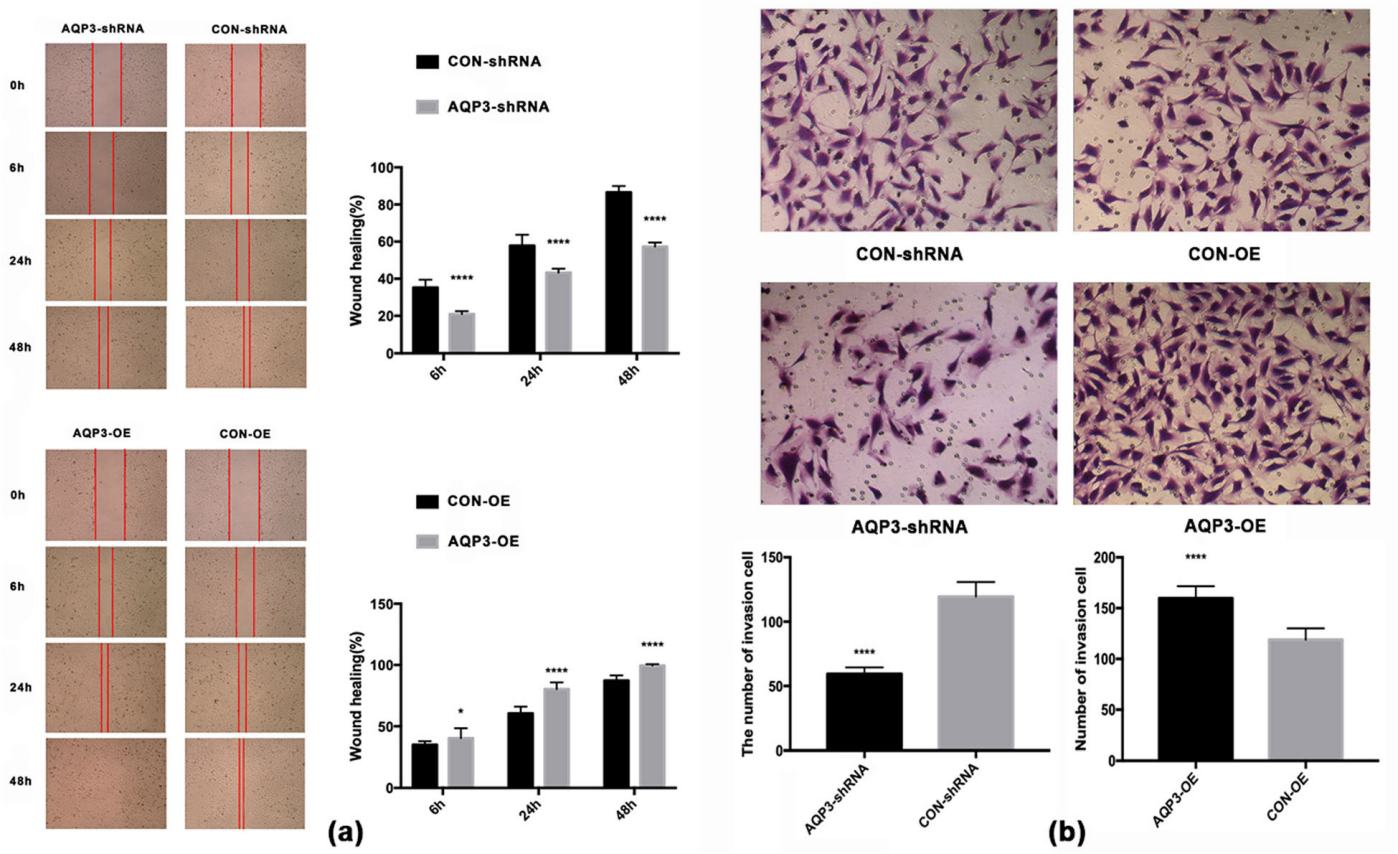
Wound healing assay and transwell assay. (**a**) Wound healing assay show the average migration rates in AQP3 knockdowned and overexpressed HTR8/SVneo (4×). (**b**) Transwell assay show the numbers of invading cells of AQP3knockdowned and overexpressed in HTR8/SVneo (10×). After AQP3 knockdown, the migratory and invasion capabilities of cells were significantly reduced, while overexpression of AQP3 significantly promoted the migration and invasion of cells

Wound healing assay show that at 6 h after scratch, cell migration rate in the AQP3-shRNA group was downregulated by 40.50% compared with the CON-shRNA group (*P* < 0.0001), while AQP3-OE group was upregulated by 15.44% compared with the CON-OE group (*P* < 0.05). At 24 h after scratch, migration rate of AQP3-shRNA was downregulated by 25.09% compared with CON-shRNA (*P* < 0.0001), while AQP3-OE group was upregulated by 32.56% compared with the CON-OE group (*P* < 0.0001). At 48 h after scratch, migration rate of AQP3-shRNA was downregulated by 33.79% compared with CON-shRNA (*P* < 0.0001), while AQP3-OE group was upregulated by 14.05% compared with the CON-OE group (*P* < 0.0001) (Table 4, Fig. 3a). After AQP3 knockdown, numbers in the AQP3-shRNA group were downregulated by 50.25% compared with the CON-shRNA group (*P* < 0.0001). After AQP3 overexpression, numbers in the AQP3-OE group were upregulated by 34.38% compared with the CON-OE group (*P* < 0.0001) (t-test), (×10 amplification) (Table 5, Fig. 3b).

**Table 4:** Migration rates of HTR8/Svneo cells

Time (after scratch)	Migration rates (Mean ± SEM)		p-value
	AQP3-shRNA	CON-shRNA	
6 h	20.96% ± 0.4032%	35.22% ± 1.099%	< 0.0001
24 h	43.29% ± 0.5446%	57.79% ± 1.531%	< 0.0001
48 h	57.32% ± 0.5719%	86.57% ± 0.8777%	< 0.0001
**Time (after scratch)**	**Migration rates (Mean ± SEM)**		**p-value**
	AQP3-OE	CON-OE	
6 h	40.35% ± 2.074%	34.95% ± 0.7426%,	< 0.05
24 h	80.31% ± 1.425%	60.59% ± 1.425%	< 0.0001
48 h	99.61% ± 0.2702%	87.34% ± 1.09%	< 0.0001

**Table 5:** Numbers of invading cells in HTR8/Svneo cells

group	invading cells (Mean ± SEM)	p-value
AQP3-shRNA	59.4 ± 1.29	< 0.0001
CON-shRNA	119.4 ± 2.952	
AQP3-OE	159.7 ± 3.046	< 0.0001
CON-OE	118.9 ± 2.914	

### Whole genome expression profile

**Fig. 4:**
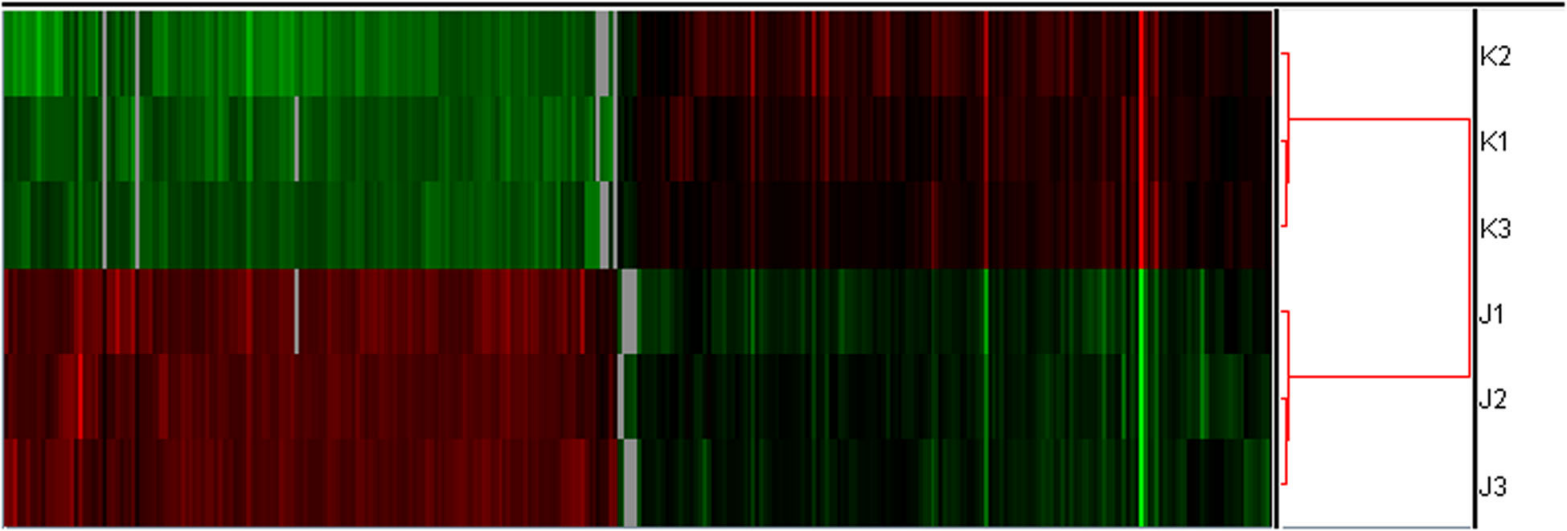
Clustering gene expression patterns. J: AQP3-shRNA, K: CON-shRNA. Red represents gene upregulation, while green represents gene downregulation

**Fig. 5:**
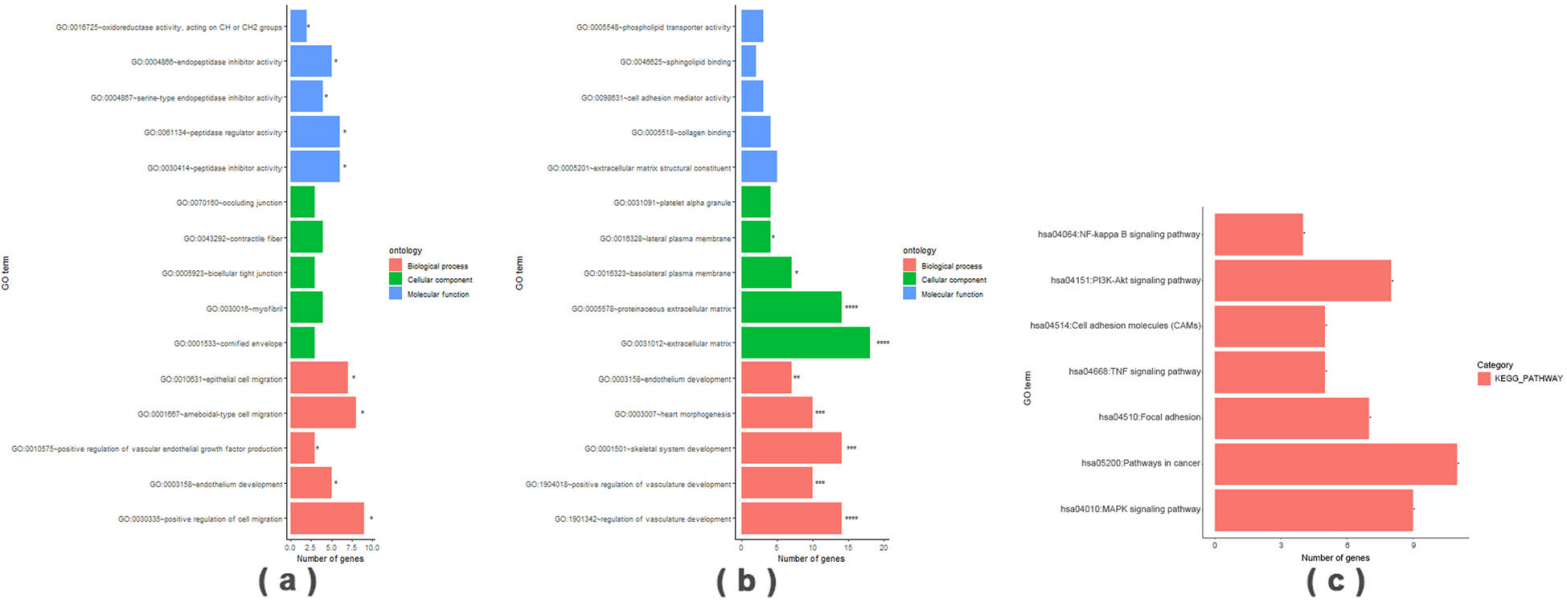
GO enrichment analysis of differentially expressed genes after AQP3 downregulation. (**a**), (**b**): Red represents result of biological enrichment, green represents result of cell component enrichment and blue represents result of molecular function enrichment. (**c**) Some entries of the Pathway on KEGG enrichment analysis of differentially expressed genes after down-regulation of AQP3 genes

**Fig. 6:**
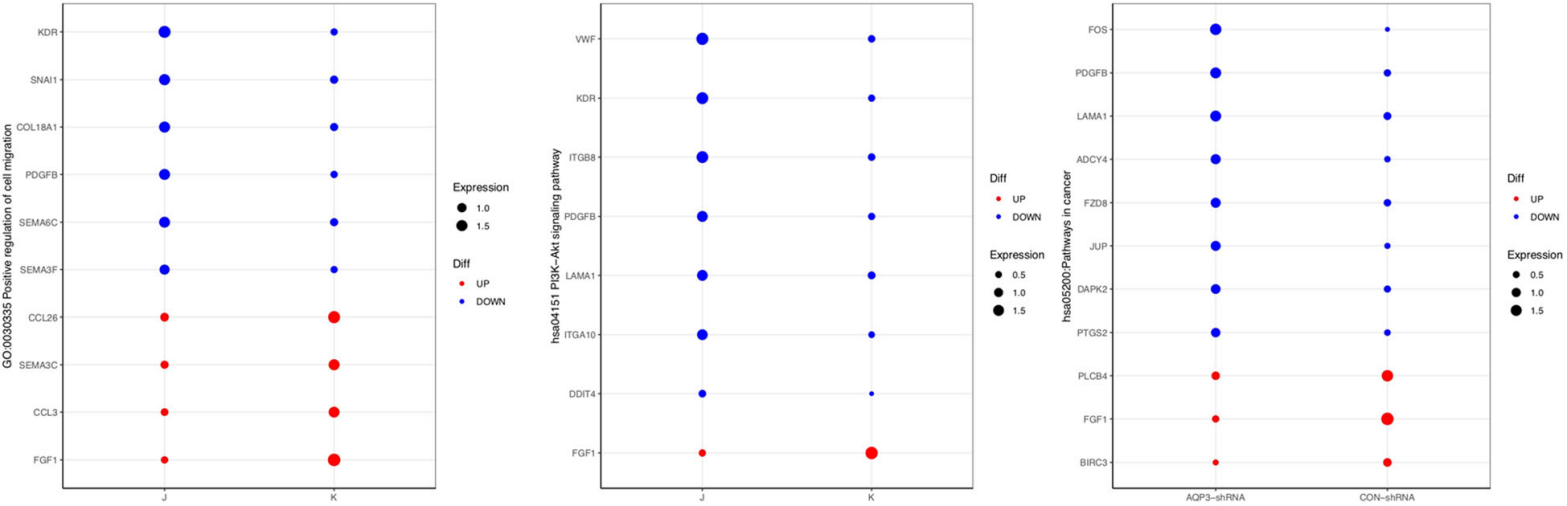
KEGG enrichment analysis of cellular pathways associated with differentially expressed genes after AQP3 downregulation. Expression abundance of genes involved in cell migration, adhesion, PIK3, and NF-κB signaling pathway J: AQP3-shRNA, K: CON-shRNA. The size of each dot represents expression abundance, while difference is shown by color: blue, down-regulated significantly; red, up-regulated significantly

To further study signaling pathways regulated by AQP3 during embryo implantation, an Agilent gene expression microarray was used to examine AQP3-shRNA and CON-shRNA groups. Genes with differential expression fold-changes≥2 and q < 0.05 as screened with Significance Analysis of Microarrays software were taken as significant differentially expressed genes. The results indicated that after AQP3 gene downregulation, there were 311 significant differentially expressed genes (150 upregulated and 161 downregulated) (Fig. 4). The result of gene ontology (GO) analysis of genes with differential expression (fold-change> 2) between AQP3 knockdown and its control group indicated these genes involved in angiogenesis, cell migration, inflammatory response, cell adhesion, and extracellular matrix recombination. Among them, 11 differentially expressed genes were related to cell migration (GO: 0030335) were notably downregulated (*P* = 0.000119), resulting in some critical factors (e.g. PDFGF-B, FOS and SNAIL1). Fourteen differentially expressed genes related to cell adhesion (GO: 0007155), such as ICAM-1, COL18A, and JUP, were significantly downregulated (*P* = 0.00345) (Fig. 5a, b; Fig. 6). Screening of differentially expressed genes by Kyoto Encyclopedia of Genes and Genomes (KEGG) enrichment analysis revealed participation primarily in tumor cell metastasis, adhesion, and apoptosis, as well as MAPK, PIK3-AKT, cell adhesion-related, tumor necrosis factor (TNF), and NF-κB signaling pathways. Among them, the majority of genes involved in cell migration, adhesion, PIK3 and NF-κB signaling pathway were down-regulated, and AQP3-shRNA was significantly down-regulated compared with CON-shRNA, with statistically significant differences (Fig. 5c, Supplementary Table). Of all these pathways, changes in cell migration and adhesion-related signaling were the most significant.

### To verify the expression of some differentially expressed genes selected from the results of whole genome expression profile

**Fig. 7:**
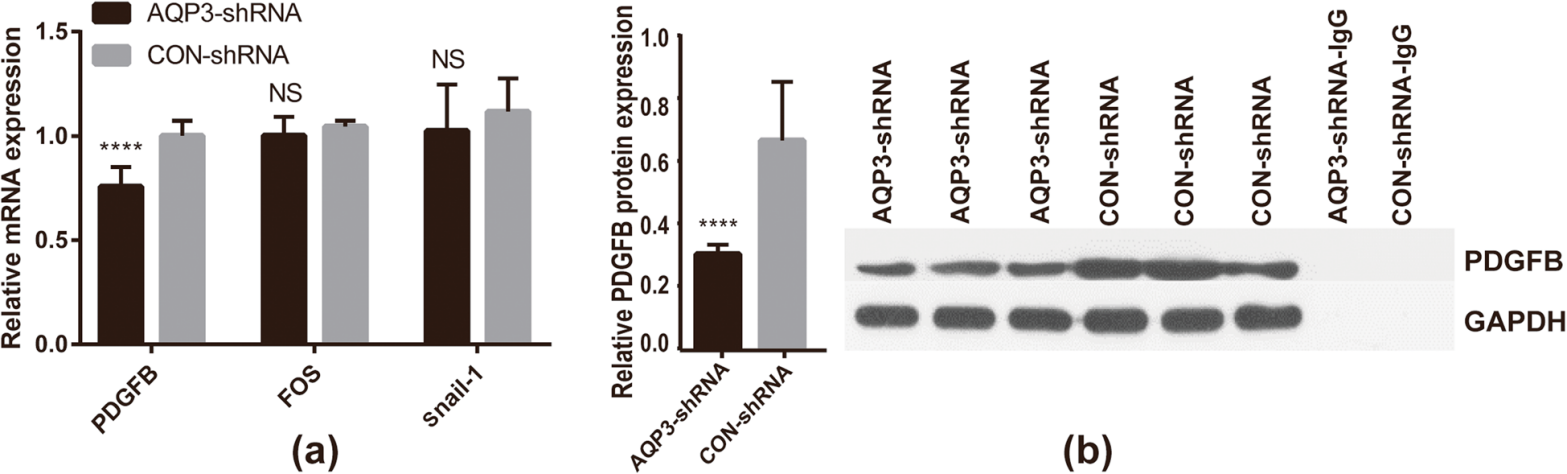
PDGF-B, FOX, Snail1 expression in HTR8/Svneo cells. (**a**) The mRNA relative expression of PDGF-B, FOX, Snail1 in the AQP3 knockdown cells. (**b**) The PDGF-B protein level in the AQP3 knockdown cells

The results of q-PCR verfication for FDGF-B,FOS and Snail1 showed that, the mRNA expression level of FDGF-B in AQP3-shRNA group was significantly lower than that in CON-shRNA group(*P* < 0.0001), while FOS, Snail1 mRNA expression were lower than that of CON-shRNA group, but there were no significant difference (*P* values were 0.068 and 0.168, respectively) (Fig. 7a). The protein level of FDGF-B was further verified, Western blot showed that the FDGF-B protein level in AQP3-shRNA group was significantly lower than that in CON-shRNA group (P < 0.0001) (Fig. 7b).

## Discussion

It is becoming increasingly evident that AQP3 is expressed in multiple malignant tumor cells [21] and participates in both tissue oncogenicity and tumor cell migration [22–26]. The invasion process of the trophoblast into the maternal endometrium and metastasis of malignant tumors both rely on cell invasion behaviors. However, mechanisms by which AQP3 influences human reproduction are not as clear as those by which malignant tumor metastasis is promoted. Currently, most investigations on embryo implantation focus on this interaction from the aspect of endometrial receptivity. A recent study [27] suggested that AQP3 might enhance endometrial receptivity to facilitate embryo implantation, whereas few investigate the embryo’s capacity for implantation. There is no study at present to clarify the molecular mechanism by which AQP3 acts on embryo to facilitate its’ implantation.

In a previous study reported expression of AQP3 was reported in four-cell, eight-cell, morula, and blastula stages of mouse embryos, with the highest expression on the membrane of blastula-stage trophoblasts [17]. Further in vitro study [18] showed that, HB-EGF could enhance AQP3 expression in blastocysts in a dose-dependent manner, thereby promoting the attachment and outgrowth of blastocyst trophoblast cells, and migth mediate its extension and migration through the regulation of HB-EGF/EGFR/ERK/AQP3 signaling pathway. However, the downstream pathway of AQP3 is not clarified.

In the present study, the human HTR8/Svneo cell line was used as model of early embryonic EVT invasion and migration to construct AQP3 knockdown and overexpression cells. The results indicate that overexpression of AQP3 significantly promoted the migration and invasion of cells, in aggreement with the results of Huang et al. [24] After AQP3 knockdown, the migratory and invasion capabilities of cells were significantly reduced, and apoptosis was significantly upregulated, supporting the results of previous reports [17, 18], Reca Alejandra et al. [28] and Xiong et al. [29] .

To explore more comprehensively the signaling mechanisms of AQP3 in promoting EVT migration, this study screened shRNA interfering AQP3 and undisturbed HTR8/SVneo cells by bioinformatics analysis gene chip expression spectrum experiment. Of the 311 differentially expressed genes screened, 150 were upregulated and 161 were downregulated. GO analysis and GO enrichment analysis found that these genes are mainly involved in angiogenesis, cell migration, inflammatory response, cell adhesion, extracellular matrix recombination. Bioinformatics analysis of gene chip expression profiling data reveals that key genes related to migration, such as platelet-derived growth factor-B (PDGF-B), Snai1, and FOS, were notably down-regulated after AQP3 knockdown expression.

Further q-PCR validation of these two key genes revealed that PDGF-B mRNA expression was prominently downregulated after knockdown expression, while Snai1, FOS mRNA expression was non-significantly downregulated. PDGFB protein levels were also observably downregulated.

PDGF is a dimeric molecule existing as homodimers or heterodimers of related polypeptide chains (A and B). PDGF-B is a member of the PDGF family, which has the ability to link with cystine and play a crucial role in development, cell proliferation, cell survival, and angiogenesi s[30]. PI3K/AKT, JNK, and PLCγ pathways were involved in the process of PDGF-B binding to PDGFRβ and inducing receptor dimerizatio n[31]. Schwenke et.a l[32]. found that PDGF-B trigger undirected motility in endometrial stromal cells, while pathway inhibitor-based studies have shown that ERK1/2, PI3K/Akt and p38 signaling are associated with chemotactic motility, whereas chemokines (PDGF-B) are mainly dependent on PI3 kinase/Akt activation. Jing et al .[33] found that PDGFB and PI3K/AKT signaling pathways have co-expression networks together with the false detection rate is very low, and PDGFB promote the metastasis of oral squamous cell carcinoma through the PI3K/AKT signaling pathway. In this study, the differentially expressed genes screened KEGG pathway analysis are mainly involved in tumor-related pathways, MAPK pathways, PI3K-AKT pathways, and NF-B signaling pathways. PI3K-AKT pathways have also been identified as closely related to tumor cell migration. Therefore, we speculate that AQP3 may play a role by acting on PDGF-B gene and PI3K/AKT signaling pathways that mediate the migration and invasion of extravillous trophoblastic cells, thereby mediating embryo implantation. However, further investigation is needed to confirm this conjecture.

## Conclusions

Collectively, these results reveal that AQP3 is an important positive regulatory factor for fetal-maternal crosstalk during the first trimester of pregnancy, whereby it may act on PDGF-B gene to promote migration and invasion ability of trophoblast cells, the underlying mechanism still requires a further investigation.

## Supplementary Information

**Additional file 1.** Supplementary table. Functional categories of selected genes differentially expressed in the HTR8/Svneo cells AQP3-shRNA and CON-shRNA.

## Data Availability

Not applicable.

## Abbreviations

AQP3: Aquaporin3
RIF: Recurrent implantation failure
EVT: Extravillous trophoblasts
EMT: Epithelial-mesenchymal transition
HB-EGF: Heparin-binding epidermal growth factor-like growth factor
