# Supplementary material for: Aquaporin 3 promotes human extravillous trophoblast migration and invasion
Source: Reprod Biol Endocrinol. 2021 Mar 29;19:49. doi: 10.1186/s12958-021-00726-z (PMC8006384; doi:10.1186/s12958-021-00726-z)
Supplement: Supplementary file 1 — Additional file 1. Supplementary table. Functional categories of selected genes differentially expressed in the HTR8/Svneo cells AQP3-shRNA and CON-shRNA. [file 12958_2021_726_MOESM1_ESM.docx]

|  | Functional category | Gene ID | Gene Name | AQP3-shRNA /CON-shRNA | |
| --- | --- | --- | --- | --- | --- |
|  |  |  |  | Fold Change | q-value(%) |
|  | Migration | A_23_P211212 | [COL18A1](http://genome-www4.stanford.edu/cgi-bin/SMD/source/sourceResult?choice=Gene&option=Name&criteria=COL18A1) | 0.4816 | 0.0000 |
|  |  | A_33_P3316273 | [CCL3](http://genome-www4.stanford.edu/cgi-bin/SMD/source/sourceResult?choice=Gene&option=Name&criteria=CCL3) | 2.2506 | 0.0000 |
|  |  | A_33_P3284919 | [SEMA6C](http://genome-www4.stanford.edu/cgi-bin/SMD/source/sourceResult?choice=Gene&option=Name&criteria=SEMA6C) | 0.4958 | 0.0000 |
|  |  | A_24_P339944 | [PDGFB](http://genome-www4.stanford.edu/cgi-bin/SMD/source/sourceResult?choice=Gene&option=Name&criteria=PDGFB) | 0.3858 | 0.0000 |
|  |  | A_33_P3813128 | [SEMA3F](http://genome-www4.stanford.edu/cgi-bin/SMD/source/sourceResult?choice=Gene&option=Name&criteria=SEMA3F) | 0.4452 | 0.0000 |
|  |  | A_23_P256473 | [SEMA3C](http://genome-www4.stanford.edu/cgi-bin/SMD/source/sourceResult?choice=Gene&option=Name&criteria=SEMA3C) | 2.0304 | 0.0000 |
|  |  | A_23_P213336 | [FGF1](http://genome-www4.stanford.edu/cgi-bin/SMD/source/sourceResult?choice=Gene&option=Name&criteria=FGF1) | 3.4933 | 0.0000 |
|  |  | A_23_P131846 | [SNAI1](http://genome-www4.stanford.edu/cgi-bin/SMD/source/sourceResult?choice=Gene&option=Name&criteria=SNAI1) | 0.4963 | 0.0000 |
|  |  | A_24_P71973 | [KDR](http://genome-www4.stanford.edu/cgi-bin/SMD/source/sourceResult?choice=Gene&option=Name&criteria=KDR) | 0.3072 | 0.0000 |
|  |  | A_23_P215484 | [CCL26](http://genome-www4.stanford.edu/cgi-bin/SMD/source/sourceResult?choice=Gene&option=Name&criteria=CCL26) | 2.1515 | 0.2300 |
|  | PIK3-Akt signaling pathway | A_32_P313405 | [LAMA1](http://genome-www4.stanford.edu/cgi-bin/SMD/source/sourceResult?choice=Gene&option=Name&criteria=LAMA1) | 0.4738 | 0.0000 |
|  |  | A_23_P105562 | [VWF](http://genome-www4.stanford.edu/cgi-bin/SMD/source/sourceResult?choice=Gene&option=Name&criteria=VWF) | 0.3174 | 0.0000 |
|  |  | A_24_P339944 | [PDGFB](http://genome-www4.stanford.edu/cgi-bin/SMD/source/sourceResult?choice=Gene&option=Name&criteria=PDGFB) | 0.3858 | 0.0000 |
|  |  | A_24_P759477 | [ITGB8](http://genome-www4.stanford.edu/cgi-bin/SMD/source/sourceResult?choice=Gene&option=Name&criteria=ITGB8) | 0.3509 | 0.0000 |
|  |  | A_33_P3263432 | [ITGA10](http://genome-www4.stanford.edu/cgi-bin/SMD/source/sourceResult?choice=Gene&option=Name&criteria=ITGA10) | 0.3219 | 0.0000 |
|  |  | A_23_P213336 | [FGF1](http://genome-www4.stanford.edu/cgi-bin/SMD/source/sourceResult?choice=Gene&option=Name&criteria=FGF1) | 3.4933 | 0.0000 |
|  |  | A_24_P71973 | [KDR](http://genome-www4.stanford.edu/cgi-bin/SMD/source/sourceResult?choice=Gene&option=Name&criteria=KDR) | 0.3072 | 0.0000 |
|  |  | A_23_P104318 | [DDIT4](http://genome-www4.stanford.edu/cgi-bin/SMD/source/sourceResult?choice=Gene&option=Name&criteria=DDIT4) | 0.2764 | 0.0000 |
|  | hsa05200:Pathways in cancer | A_23_P501822 | [JUP](http://genome-www4.stanford.edu/cgi-bin/SMD/source/sourceResult?choice=Gene&option=Name&criteria=JUP) | 0.3045 | 0.0000 |
|  |  | A_23_P396858 | [FZD8](http://genome-www4.stanford.edu/cgi-bin/SMD/source/sourceResult?choice=Gene&option=Name&criteria=FZD8) | 0.4858 | 0.0000 |
|  |  | A_32_P313405 | [LAMA1](http://genome-www4.stanford.edu/cgi-bin/SMD/source/sourceResult?choice=Gene&option=Name&criteria=LAMA1) | 0.4738 | 0.0000 |
|  |  | A_23_P381261 | [ADCY4](http://genome-www4.stanford.edu/cgi-bin/SMD/source/sourceResult?choice=Gene&option=Name&criteria=ADCY4) | 0.3363 | 0.0000 |
|  |  | A_23_P106194 | [FOS](http://genome-www4.stanford.edu/cgi-bin/SMD/source/sourceResult?choice=Gene&option=Name&criteria=FOS) | 0.1205 | 0.0000 |
|  |  | A_33_P3260634 | [PLCB4](http://genome-www4.stanford.edu/cgi-bin/SMD/source/sourceResult?choice=Gene&option=Name&criteria=PLCB4) | 2.0091 | 0.0000 |
|  |  | A_24_P339944 | [PDGFB](http://genome-www4.stanford.edu/cgi-bin/SMD/source/sourceResult?choice=Gene&option=Name&criteria=PDGFB) | 0.3858 | 0.0000 |
|  |  | A_24_P250922 | [PTGS2](http://genome-www4.stanford.edu/cgi-bin/SMD/source/sourceResult?choice=Gene&option=Name&criteria=PTGS2) | 0.4157 | 0.0000 |
|  |  | A_23_P98350 | [BIRC3](http://genome-www4.stanford.edu/cgi-bin/SMD/source/sourceResult?choice=Gene&option=Name&criteria=BIRC3) | 2.3975 | 0.0000 |
|  |  | A_23_P213336 | [FGF1](http://genome-www4.stanford.edu/cgi-bin/SMD/source/sourceResult?choice=Gene&option=Name&criteria=FGF1) | 3.4933 | 0.0000 |
|  |  | A_24_P10233 | [DAPK2](http://genome-www4.stanford.edu/cgi-bin/SMD/source/sourceResult?choice=Gene&option=Name&criteria=DAPK2) | 0.4678 | 0.0000 |
|  |  |  |  |  |  |
|  |  |  |  |  |  |
